# Nicotinamide and Pyridoxine Supplementation Enhances Muscle Stem Cell Activity and Muscle Regeneration in Humans: A Randomized Placebo‐Controlled Clinical Trial of High Force Eccentric Contraction Recovery in Healthy Young Men

**DOI:** 10.1002/advs.202518471

**Published:** 2026-03-24

**Authors:** Grith Højfeldt, Joris Michaud, Ann Damgaard, Karoline Karlog, Eugenia Migliavacca, Sonia Karaz, Elham Pazirandeh‐Micol, Odd E. Johansen, Leonidas G. Karagounis, Bjørk W. Helge, William Hagemann, Michael Kjaer, Jerome N. Feige, Pascal Stuelsatz, Abigail L. Mackey

**Affiliations:** ^1^ Institute of Sports Medicine Copenhagen Department of Orthopaedic Surgery Copenhagen University Hospital – Bispebjerg and Frederiksberg Copenhagen Denmark; ^2^ Department of Clinical Medicine Faculty of Health and Medical Sciences University of Copenhagen Denmark; ^3^ Nestlé Institute of Health Sciences Nestlé Research Lausanne Switzerland; ^4^ Clinical Research Unit Nestlé Research Lausanne Switzerland; ^5^ Translational Research Nestlé Health Science Vevey Switzerland; ^6^ Mary MacKillop Institute for Health Research (MMIHR) Australian Catholic University Melbourne Australia; ^7^ Institute of Social and Preventive Medicine (ISPM) University of Bern Bern Switzerland

**Keywords:** human muscle regeneration, muscle stem cell, muscle therapeutics, nicotinamide, pyridoxine, randomized placebo‐controlled clinical trial

## Abstract

Muscle Stem Cells (MuSCs) drive muscle regeneration and slow pathological progression of muscle diseases. In preclinical models, nicotinamide (NAM) and pyridoxine (PN) synergistically increased MuSC proliferation and differentiation, and accelerated muscle regeneration. Herein we tested if NAM/PN could enhance MuSC activity and muscle regeneration in a randomized, placebo‐controlled clinical trial. Men aged 18–49 years were supplemented daily with 714 mg NAM and 19 mg PN, or placebo, for 9 days following one session of damaging unilateral eccentric muscle contractions. The primary endpoint was MuSC activity via immunohistofluorescence on biopsy sections from the vastus lateralis muscle. Histological markers of muscle regeneration constituted secondary outcomes, and muscle damage was validated with clinical markers. 39 out of 43 enrolled participants completed the study. Supplementation of NAM/PN was well tolerated and increased blood concentrations of NAM and PN vitamers. 8 days after the contraction protocol, the number of Pax7, MyoD, and myogenin positive cells per damaged fiber was significantly higher in NAM/PN vs placebo groups (+29%–67%). NAM/PN also increased the proportion of regenerating fibers (+37%). Daily oral NAM/PN supplementation after high intensity muscle contractions enhances MuSC activity and accelerates muscle regeneration and repair, providing new opportunities for therapeutic applications in muscle recovery and muscle wasting disorders.

## Graphical abstract

In a randomized clinical trial, we test the potential of combined nicotinamide (NAM) and pyridoxine (PN) to improve muscle recovery through muscle stem cell (MuSC) activity. Daily oral NAM and PN supplementation after high intensity muscle contractions enhances MuSC activation and differentiation, and accelerates muscle regeneration, providing new opportunities for therapeutic applications in muscle recovery and muscle wasting disorders.

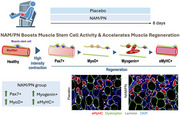

## 1 Introduction

Muscle stem cells (MuSCs), the resident stem cells of skeletal muscle tissue, primarily drive muscle regeneration during repair following myofiber injury or muscle diseases [1], and actively contribute to muscle growth during early life [2, 3]. MuSCs have also been proposed to contribute to myonuclear maintenance during muscle homeostasis throughout life [4, 5, 6], although this remains under discussion [7, 8]. Nevertheless, in adaptive contexts, MuSCs fuse with myofibers to regulate myonuclear domains and facilitate resistance training–induced skeletal muscle hypertrophy [9, 10, 11, 12]. Under homeostatic conditions, the majority of MuSCs remain in a quiescent state. Upon activation by mechanical or inflammatory signals from the niche in response to contraction, injury or pathology, MuSCs proliferate, differentiate, and either fuse with existing myofibers or with each other to replace necrotic myofibers. They can also self‐renew to replenish the MuSC pool. At the molecular level, MuSCs and their progeny are tightly regulated by sequential expression of transcription factors, notably Pax7 and the myogenic regulatory factors MyoD and myogenin, allowing for monitoring MuSC progression [13].

Maintaining healthy skeletal muscle is a major determinant of the quality of life across the lifespan and MuSCs play a crucial role in preserving its regenerative and adaptive capacity [14]. Skeletal muscle injuries are common occurrences and are frequently observed in both recreational and professional sports as a result of strains and contusions [15, 16]. Strenuous and unaccustomed exercise, especially exercise involving eccentric muscle contractions where a muscle lengthens while generating force, induce damage to the cellular structures and the extracellular matrix [17]. This contraction mode is typically involved in exercises where the muscle is actively resisting the external load or gravity, such as weightlifting, downhill running, jumping, and squatting. Other causes of muscle injury include laceration resulting from accidents during activities of daily living and in the workplace, or iatrogenic muscle damage following surgical procedures [17]. While skeletal muscle possesses the ability to recover from tissue damage, the process of muscle regeneration is often slow, extending beyond 4 weeks in humans [18], hindering the return to regular activities. Moreover, a range of conditions such as cachexia [19, 20, 21], diabetes [22, 23], and muscular dystrophies [22, 23], disrupt myofiber integrity and impair the muscle's natural ability to regenerate and repair. Aging exerts similar defects in rodents [24], while humans appear less affected [25]. Despite the high prevalence of muscle injuries, traditional therapeutic options for treating damaged muscles are limited and include variations on RICE (Rest, Ice, Compression and Elevation), POLICE (Protection, Optimal Loading, Ice, Compression and Elevation) [26] and more recently PEACE and LOVE (Protection, Elevation, Avoid Anti‐inflammation, Compression, Education & Load, Optimism, Vascularization and Exercise) protocols, followed by physical therapy and rehabilitation exercises [27]. The use of nonsteroidal anti‐inflammatory drugs (NSAIDs) shortly after a muscle injury can provide pain relief and reduce inflammation and swelling [28]. However, there are conflicting data regarding their long‐term effects on muscle regeneration [29]. Some studies suggest that NSAIDs may offer little to no benefit in promoting muscle healing and may even have detrimental effects that delay the recovery process [30], and the current PEACE and LOVE guidelines advise against NSAID use. Importantly, there is a lack of therapeutic interventions that effectively support muscle recovery by directly targeting the endogenous regenerative potential of skeletal muscle.

In a previous study, we used a high‐content imaging screen of a library of over 50 000 natural bioactive molecules and food‐derived nutrients on human myogenic progenitors to identify novel nutritional molecules targeting MuSCs [31]. Using this approach, we discovered that nicotinamide (NAM) and pyridoxine are potent nutrients that stimulate MuSC proliferation and induce their differentiation. We then tested the efficacy of oral NAM/PN supplementation in two different preclinical models of muscle regeneration, after an acute muscle injury induced with cardiotoxin and in a model of eccentric contraction‐induced muscle regeneration. Based on our collective findings, NAM/PN has emerged as a promising nutritional intervention that stimulates MuSCs and enhances muscle repair, with potential for direct translation into clinical applications.

While preclinical models of muscle regeneration are well‐established [32], studying muscle regeneration in humans is more complex as it requires a standardization of the complex physiological and molecular aspects that drive muscle repair and recovery. Human models of muscle recovery, typically exercise‐based, often exhibit high inter‐individual variability and may not always result in myofiber necrosis, indicating more of a muscle remodeling process rather than true regeneration. To overcome this limitation, we have developed a technique that combines neuromuscular electrical muscle stimulation (NMES) with forced lengthening contractions in a controlled setting [25, 29, 33, 34, 35]. This approach induces myofiber necrosis followed by muscle regeneration with full restoration of force producing capacity and no long‐term side effects. Notably, while most other models succeed at triggering the initial phase of muscle regeneration as indicated by the expansion of Pax7^+^ MuSCs, a robust increase in differentiating progenies (myogenin^+^ cells and eMyHC^+^ fibers) has been observed primarily with this NMES protocol [29, 34, 36, 37, 38].

We hypothesized that the NAM/PN nutritional intervention, already established to enhance muscle repair in rodents, would also improve MuSC‐mediated repair in a human model of muscle regeneration following high force eccentric contraction. To this end, we performed a randomized, double‐blind, placebo‐controlled clinical trial comparing an oral NAM/PN supplementation to placebo during recovery from a NMES eccentric contraction protocol in heathy young men. The pre‐registered primary outcome was the number of MuSCs (Pax7^+^ and myogenin^+^) quantified via immunohistofluorescence on muscle biopsy sections. Secondary endpoints included further histological markers of muscle regeneration, and clinical signs of muscle damage were assessed as exploratory outcomes to validate and monitor inter‐patient responses to the NMES protocol.

## 2 Results

### 2.1 Description of the Study

**FIGURE 1:**
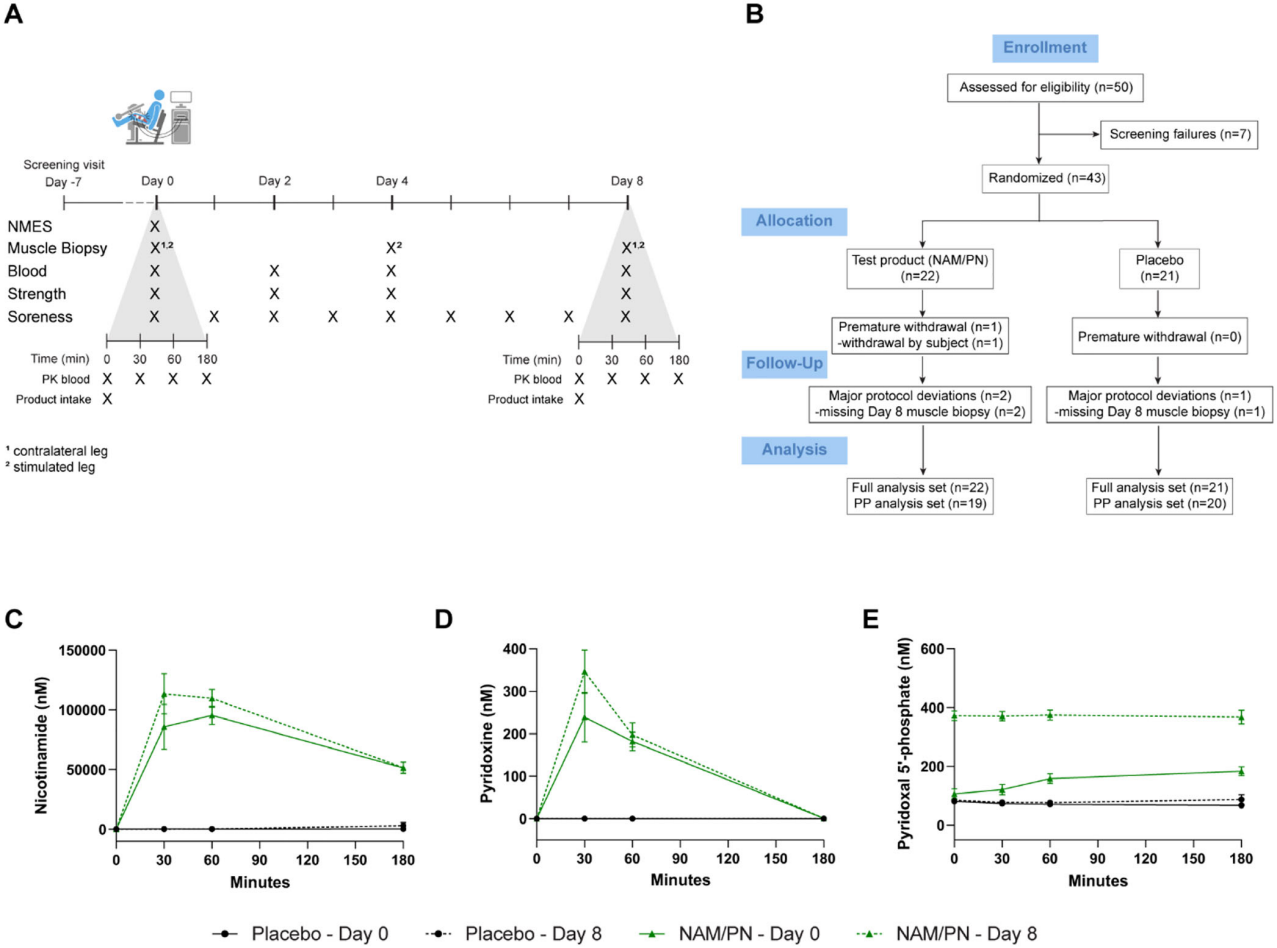
Study design and NAM/PN pharmacokinetic. (A) Overview of the study design indicating the timing of muscle biopsies, blood samplings, measurements of muscle strength and soreness during the 9 days (from Day 0 to Day 8) period of NAM/PN or placebo intake in healthy male participants. The bottom timeline displays in more detail the timing and order of events during the 3 h protocol at Day 0 when Neuromuscular electrical stimulation (NMES) was performed. (B) CONSORT flow chart. (C–E) Pharmacokinetic profiles of (C) nicotinamide, (D) pyridoxine and (E) pyridoxal 5’‐phosphate (bioactive form of pyridoxine), were assessed on blood samples at Day 0 and Day 8. For each day, blood has been collected at four different timepoints (pre‐supplementation and 30 min, 1 h, and 3 h post‐supplementation). NAM/PN group, n = 21 and 20 at Day 0 and Day 8, respectively; placebo group, n = 21 and 20 at Day 0 and Day 8, respectively. Data are represented as mean ± SEM.

The primary objective of this study was to investigate if a combination of nicotinamide (NAM) and pyridoxine (PN) intake improves human skeletal muscle regeneration, through enhanced activation of MuSCs. To this end, the primary endpoint was the number of MuSCs measured by immunofluorescence on sections of muscle biopsies during experimentally induced muscle regeneration. An overview of the study design and study flow diagram are presented in Figure 1A,B. Forty‐three healthy males aged 18 to 49 years were enrolled and randomly assigned to supplementation with either NAM/PN (NAM: 714 mg/day, PN: 19 mg/day, n = 22) or placebo (n = 21) (Figure 1B, see Table 1 for an overview of participant characteristics). Although the inclusion criteria allowed participants aged 18–50 years, the actual age distribution was much narrower, with only a few individuals older than 35 and a mean age of 26.7 years (SEM = 1.22; Figure S1A). This limited age range reduces the likelihood that age‐related variability influenced the MuSC response. One participant prematurely withdrew, and three participants had a major protocol deviation independent of the treatment (two missed the last visit and one Day 8 muscle biopsy was not usable). Thus, 39 participants completed the study (19 NAM/PN, 20 placebo).

**TABLE 1:** Population demographics & pehnotypes at baseline.


	Number of participants (% total) or mean (SD)		
Characteristic	Overall	Test product	Placebo
Age (Years)	27.1 (7.7)	27.0 (8.1)	27.2 (7.5)
Body Mass Index (kg/m2)	23.7 (2.6)	23.6 (2.5)	23.8 (2.7)
**Stimulated Leg**			
Left	22 (51.2%)	13 (59.1%)	9 (42.9%)
Right	21 (48.8%)	9 (40.9%)	12 (57.1%)
Dominant	20 (46.5%)	11 (50.0%)	9 (42.9%)
Non‐Dominant	23 (53.5%)	11 (50.0%)	12 (57.1%)

Data are for the Full analysis set (FAS). SD = standard deviation; % = percent.

The NAM/PN supplementation was well tolerated, and no adverse events related to the NAM/PN supplementation were reported. Overall, a total of 15 mild adverse events and no serious adverse events were reported (Table 2). One participant contracted a COVID‐19 infection during the trial and missed the last visit on Day 8. Other mild adverse events were related to muscle injury or muscle biopsy procedures and all were resolved rapidly. NAM/PN supplementation significantly increased serum levels of NAM and PN confirming compliance and systemic bioavailability of the supplement (Figure 1C–E). Levels of NAM and PN were similar after the first and last dose. The bioactive form of PN, pyridoxal‐5’‐phosphate (PLP) was significantly higher on day 8 than after the first dose on day 0 (Figure 1E), demonstrating that PN was effectively processed and bioconverted during the supplementation. Importantly, before starting supplementation, basal plasma levels of nicotinamide and pyridoxine (PLP) were above the deficiency threshold for all participants (Figure S1B,C).

**TABLE 2:** List of adverse events (full analysis set).


Type of adverse event	Events in test product group	Events in placebo group	Severity	Relationship to NAM/PN supplementation
**All adverse events**	9	6		
**Serious adverse events**	0	0		
**Infections and infestations**	1	0		
‐ COVID‐19	1	0	Mild	Not Related
**Injury, poisoning and procedural complications**	1	1		
‐ Anaesthetic complication	1	1	Mild	Not Related
‐ Musculoskeletal procedural complication	1	1	Mild	Not Related
**Investigations**	4	1		
‐ Aspartate aminotransferase increased	4	1	Mild	Not Related
**Musculoskeletal and connective tissue disorders**	2	1		
‐ Pain in extremity	2	1	Mild	Not Related
**Nervous system disorders**	1	2		
‐ Headache	1	2	Mild	Not Related
**Renal and urinary disorders**	0	1		
‐ Haematuria	0	1	Mild	Not Related

### 2.2 Assessment of Muscle Damage and Its Link to Regeneration

The NMES protocol used in this study is known to induce significant muscle damage [36]. Measuring the extent of muscle damage is essential to evaluate the efficiency of the regenerative response as the activation of MuSCs and the initiation of a regenerative response are directly triggered by the presence of muscle damage, and their amplitude depends on the extent of the damage.

**FIGURE 2:**
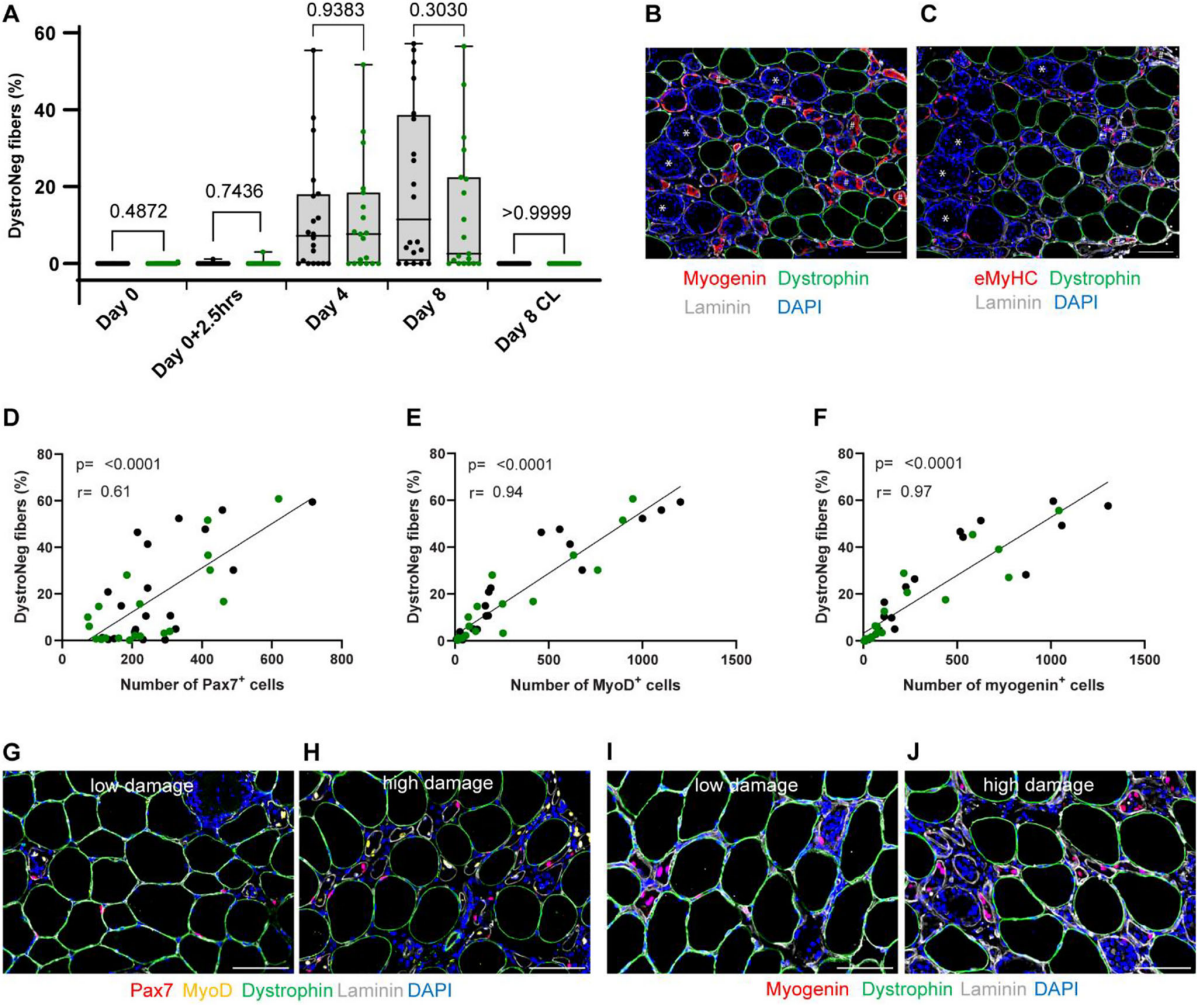
Assessment of muscle damage and its link to regeneration. (A) Muscle damage was assessed by the presence of dystrophin‐negative (DystroNeg) fibers on cross sections of muscle biopsies collected at baseline (Day 0, contralateral leg) and after having induced a muscle damage at Day 0 (Day 0 + 2.5 h, stimulated leg), Day 4 (stimulated leg) and Day 8 (stimulated and contralateral legs). Data are expressed as the percentage of dystrophin‐negative fibers out of total fibers. (B,C) Representative images illustrating the massive infiltration of nuclei inside dystrophin‐negative fibers undergoing inflammatory infiltration and necrosis (examples shown by *), and the overlap with active regeneration demonstrated marked by myogenin^+^ nuclei and eMyHC‐positive fibers (examples shown by #). (D–F) Correlation analyses between the percentage of dystrophin‐negative fibers and the number of (D) Pax7^+^ cells, (E) MyoD^+^ cells and (F) myogenin^+^ cells. (G–J) Representative images of a muscle biopsy with low and high levels of muscle damage are shown in (G,I) and (H,J), respectively. Individual dots represent individual participants from the Placebo group (black dots, n = 20) and the NAM/PN group (green dots, n = 19). Scale bars = 100 µm.

To ensure the proper execution of this NMES protocol, classical clinical signs of muscle damage were first assessed. All groups exhibited increased muscle soreness, elevated circulating creatine kinase levels, and reduced quadriceps muscle strength with no effect of the supplementation on these parameters (Figure S1D–F). To further quantify the extent of muscle damage, we analyzed muscle biopsy cross‐sections for dystrophin‐negative fibers as myofiber damage and necrosis is well described to cause loss of dystrophin expression in humans [35] (Figure 2). The first significant appearance of such dystrophin‐negative fibers was observed at Day 4 with a further increase at Day 8 (Figure 2A). As shown in previous studies using the same model, dystrophin‐negative fibers are reliable indicators of muscle damage, as they are typically associated with the loss of other structural proteins, macrophage infiltration, and markers of regeneration [25, 29, 35, 36, 39]. In the present biopsies, these fibers displayed either extensive infiltration of nuclei, indicative of ongoing necrosis, or expressed myogenin and eMyHC, both hallmarks of active regeneration (Figure 2B,C).

The proportion of dystrophin‐negative fibers at Day 8 did not differ significantly between the placebo vs. NAM/PN groups. The amount of dystrophin‐negative fibers was highly variable between participants, ranging from 0 to ∼60% of total fibers (Figure 2A). As expected, the extent of stem cell responses was closely linked to the severity of muscle damage (Figure 2D–J). The percentage of dystrophin‐negative fibers strongly correlated with the number of Pax7^+^ cells (Figure 2D, r = 0.61), MyoD^+^ cells (Figure 2E, r = 0.94), and myogenin^+^ cells (Figure 2F, r = 0.97). These results confirm the existence of a direct relationship between the extent of muscle damage and the amplitudes of MuSC activation and regenerative response. In order to compare the regenerative capacity between individuals, MuSC outcomes such as Pax7^+^, MyoD^+^, and myogenin^+^ were adjusted for the extent of muscle damage measured on the same histological section by normalizing these outcomes to the number of dystrophin‐negative fibers (Figure 2G–J). This approach also minimizes variability due to sampling differences between biopsies, which may not always reflect the overall condition of the entire muscle. By assessing both muscle damage and MuSC activation on the same tissue section, we hence ensured a more accurate and internally controlled comparison of regenerative responses across individuals.

### 2.3 NAM/PN Supplementation Increases the Amplification and Differentiation of MuSCs

Muscle regeneration was induced unilaterally through repeated neuromuscular electrical stimulations (NMES) combined with eccentric contractions of the vastus lateralis muscle. While this protocol is well accepted to induce a regenerative response, only a limited number of studies have explored the kinetics of MuSC activation and differentiation during human muscle regeneration. These studies have demonstrated that MuSCs exhibit active amplification and differentiation approximately 7 to 8 days after muscle injury, with minimal to no activity detected between the first and fourth day [29, 34, 36, 37]. To overcome the limited availability of kinetic characterization of muscle stem cell fate transitions during the first week of muscle regeneration, we analyzed the most optimal time point to read out efficacy prior to unblinding of the treatment effect. In line with previous findings, our analysis of the blinded quantification of MuSC by immunohistofluorescence revealed that no MuSC amplification had occurred at Day 4 (Figure S2A–C). A slight unexpected decrease in the number of Pax7^+^ cells was detected compared to baseline, possibly corresponding to the decline of Pax7 expression after MuSC activation that has been reported in animal models [40, 41], or, alternatively, reflecting fusion of satellite cells to muscle fibers without prior proliferation as recently demonstrated [42]. A robust MuSC response became evident at Day 8, where the number of Pax7^+^ cells (Figure S2A) and myogenin^+^ cells (Figure S2B) significantly increased compared to the baseline. Furthermore, the appearance of regenerating fibers, indicated by the expression of embryonic myosin (eMyHC, Figure S2C), was also observed at Day 8 and confirmed the regenerative response.

**FIGURE 3:**
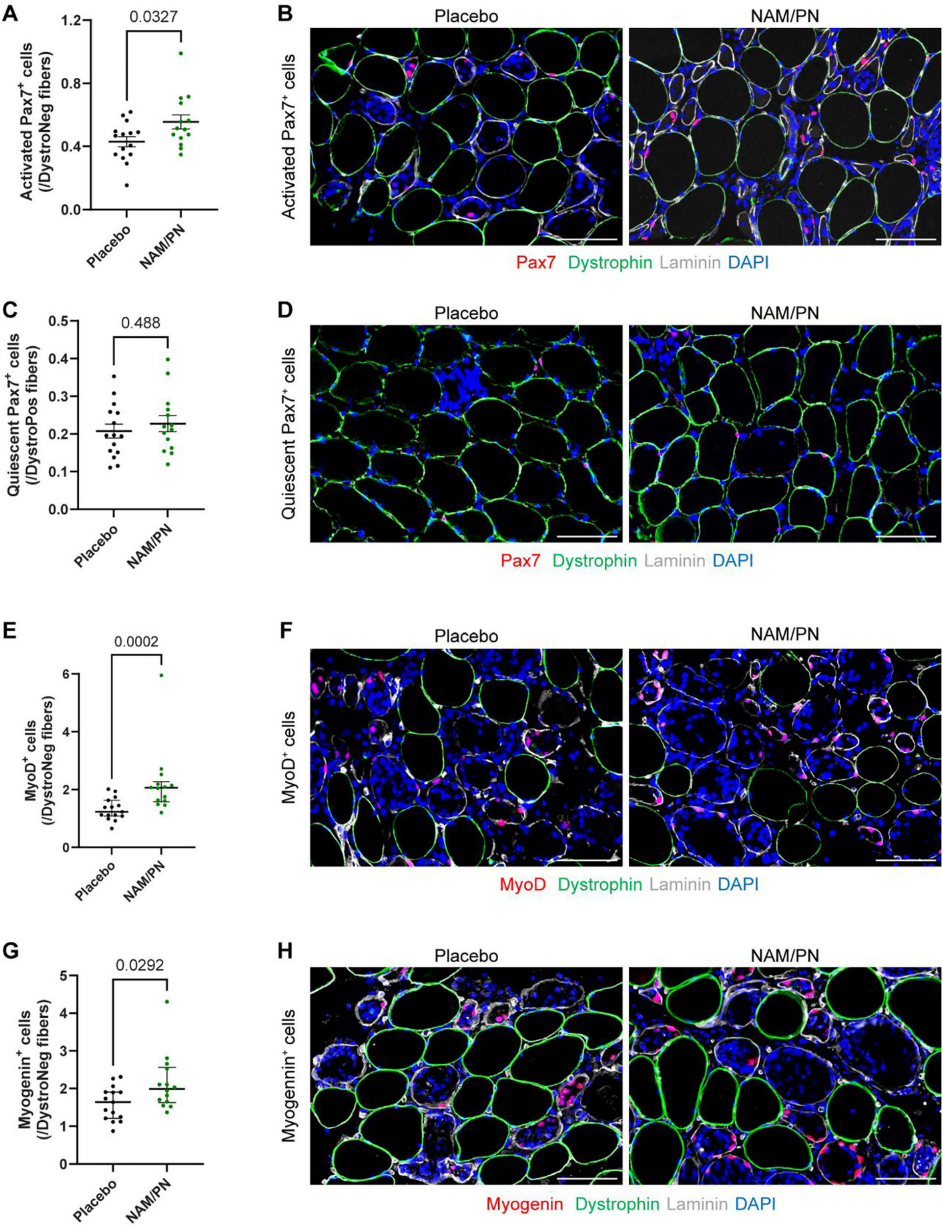
NAM/PN supplementation increases the number of activated Pax7+ cells as well as the number of MyoD+ and myogenin+ cells. The number of MuSCs and their progenies were quantified by immunohistofluorescence on cross section of biopsies collected at Day 8 (stimulated leg) and compared between NAM/PN vs. placebo group. Quantification and representative images of (A,B) activated Pax7+ cells; (C,D) quiescent Pax7+ cells; (E,F) MyoD+ cells, and (G,H) myogenin+ cells. Data are represented as (A,C) mean ± SEM or (E,G) median with interquartile range; and are expressed relative to the number of (C) dystrophin‐positive (DystroPos) and to the number of (A,E,G) dystrophin‐negative (DystroNeg) fibers. All participants with apparent muscle damage (above the first quartile based on the percentage of dystrophin‐negative fibers) on muscle biopsy at Day 8 (stimulated leg) were included in the analyses. Placebo group, n = 15; NAM/PN group, n = 14. Scale bars = 100 µm.

Given this observed regenerative timeline, we focused our subsequent analyses on Day 8 (Figure 3). To capture the main transitions from activation, proliferation up to terminal differentiation of MuSCs, we quantified the number of Pax7^+^, MyoD^+^ and myogenin^+^ cells, and distinguished activated Pax7^+^ cells surrounding a regenerating fiber from quiescent Pax7^+^ in their native niche around an uninjured fiber (Figure S3A). This classification was validated by the very high correlation between muscle damage and activated Pax7^+^ MuSCs (Figure S3B, r = 0.96; p<0.001), but not quiescent Pax7^+^ MuSCs (Figure S3C, r = −0.23; non‐significant).

The supplementation of NAM/PN significantly increased the number of activated Pax7^+^ cells (Figure 3A,B; +29%; p = 0.03 vs placebo), while the number of quiescent Pax7^+^ cells was not affected by the treatment (Figure 3C,D). Consistently, the number of MyoD^+^ cells representing differentiating MuSCs was very robustly increased following NAM/PN vs placebo supplementation (Figure 3E,F; +67%; p = 0.0002). The supplementation of NAM/PN also significantly enhanced more terminal markers of myogenic differentiation as the number of myogenin^+^ cells was also significantly elevated in NAM/PN vs placebo compared to the placebo group (Figure 3G,H; 34%; p = 0.029). While the small number of participants over 35 years old (three per group) precluded a meaningful statistical comparison between younger and older subgroups, we did not observe any apparent differences in MuSC responses between participants below and above 35 years of age. The activation of Pax7^+^, MyoD^+^ and Myogenin^+^ cells in the NAM/PN group compared to placebo was confirmed through independent statistical stratifications. These included analyses of the full participant cohort, irrespective of the degree of apparent muscle damage (FigureS3D–G; p < 0.05). These conclusions were also confirmed in exploratory analyses with subsets of participants showing 5% or more damaged fibers and those above the median value for damaged fibers (Figure S3H–O; p < 0.06). The synergistic myogenic effects of NAM and PN were originally discovered through a cellular screen in human primary myogenic progenitors where NAM primarily activated Pax7 and PN triggered the commitment to MyoD [31]. Consistently, the enhanced regenerative response upon NAM/PN supplementation in this clinical study involved a coordinated activation of the myogenic program where Pax7 activation, MyoD induction and engagement to terminally‐differentiated Myogenin‐positive progenitors were all consistently improved by the treatment. Correlation analyses demonstrated that this integrated myogenic response was at play in each participant and scaled with the extent of muscle damage as the number of activated Pax7^+^, MyoD^+^ and myogenin^+^ cells strongly correlated with each other (FigureS3P–R). Overall, our findings demonstrate that NAM/PN supplementation does not affect quiescent MuSCs but promotes the activation, proliferation, and differentiation of MuSCs by increasing the number of activated Pax7^+^ cells, MyoD^+^ cells, and myogenin^+^ cells. This demonstrates that NAM/PN supplementation facilitates the complete progression of different MuSC progenies through the myogenic program without affecting the quiescent MuSC pool.

### 2.4 NAM/PN Supplementation Accelerates Muscle Repair

**FIGURE 4:**
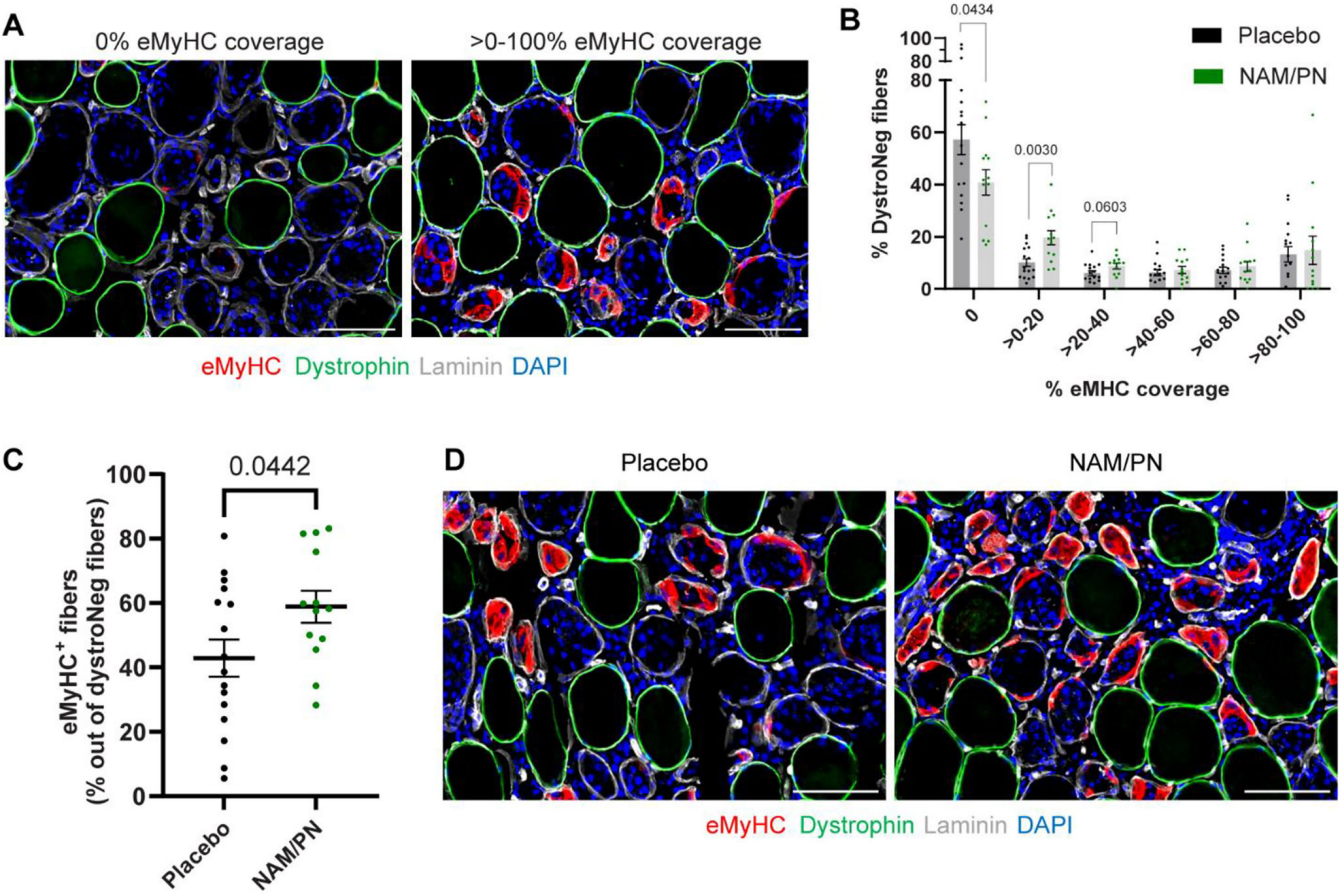
NAM/PN supplementation accelerates the muscle repair process. Fiber regeneration has been evaluated by immunohistofluorescence on cross section of biopsies collected at Day 8 (stimulated leg). (A) Representative images for eMyHC immunolabeling depicting examples of damaged fibers with different extent of eMyHC immunolabeling ranging from 0% to 100% coverage. (B) Quantification of the area covered by eMyHC immunolabeling within each damaged fiber and distribution of damaged fibers based on the percentage of eMyHC coverage. Data are represented as mean ± SEM. (C) The numbers of damaged fibers displaying immunoreactivity for embryonic myosin (eMyHC) were quantified and compared between NAM/PN vs. placebo group. Data are represented as mean ± SEM and are expressed relative to the number of dystrophin‐negative fibers. (D) Representative images for eMyHC immunolabeling illustrating difference between NAM/PN and placebo groups. (A‐D) All participants with apparent muscle damage (above the first quartile based on the percentage of dystrophin‐negative fibers) on muscle biopsy at Day 8 (stimulated leg) were included in the analyses. Placebo group, n = 16; NAM/PN group, n = 13. Scale bars = 100 µm. Individual dots represent individual participants from the placebo group (black dots) and the NAM/PN group (green dots).

The impact of NAM/PN supplementation on muscle fiber regeneration was assessed by quantifying the expression of embryonic myosin (eMyHC), a well‐accepted marker of regenerating fibers [43]. In order to assess the maturity of regenerating myofibers, damaged fibers were segmented based on the coverage of eMyHC immunolabeling, ranging from 0% to 100% of the myofiber area (Figure 4A). Analysis of the distribution profile revealed that approximately 50% of the damaged fibers did not express eMyHC (Figure 4B), which is consistent with the relatively early time point analyzed (Day 8 after injury) and previously published findings [33]. While quantification of myofiber size at this stage did not reveal significant differences between the placebo and NAM/PN group (Figure S4A), the proportion of damaged fibers lacking eMyHC expression (0% eMyHC coverage) was reduced in the NAM/PN group compared to placebo, whereas the proportion of fibers beginning to express eMyHC (>0%–20% and >20%–40% eMyHC coverage) was increased (Figure 4B). Importantly, the percentage of damaged fibers expressing eMyHC^+^ at Day 8 was significantly higher in the NAM/PN group compared to placebo (Figure 4C,D; +37%; p = 0.04), indicating that NAM/PN supplementation accelerates the differentiation of MuSCs into eMyHC^+^ regenerating fibers. Similarly to the MuSC readouts, the percentage of eMyHC^+^ fibers was associated with the extent of muscle damage (Figure S4B) and the number of eMyHC^+^ fibers strongly correlated with Pax7^+^, MyoD^+^, and myogenin^+^ cells (Figure S4C–E), confirming that increased MuSC activation upon NAM/PN treatment directly links with enhanced regeneration. Collectively, our data demonstrate that NAM/PN supplementation accelerates the muscle repair process, as evidenced by the increased proportion of damaged fibers undergoing active regeneration.

## 3 Discussion

In this study, we conducted a double‐blinded randomized placebo‐controlled clinical trial to investigate the efficacy of NAM/PN supplementation on muscle repair after eccentric contraction‐induced muscle damage in healthy men. We consistently observed improvements in the different phases of myogenesis and muscle regeneration, including the amplification of Pax7^+^ activated MuSCs, their transition to MyoD expressing progenitors, and their terminal differentiation to myogenin. Additionally, we observed a positive effect on the maturation of these myogenic progenitors into regenerating myofibers expressing embryonic myosin. This study also confirmed the tolerability and pharmacokinetics of NAM/PN supplementation in humans. The doses of NAM and PN used were within the safety limits defined by the Council for Responsible Nutrition [44]. Daily oral supplementation with NAM/PN for 9 days (from Day 0 to Day 8) did not result in any adverse effects and led to a rapid increase in serum levels of NAM and PN, with C_max_ values reached between 30 to 60 min. Interestingly, the bioactive form of PN, pyridoxal‐5’‐phosphate (PLP), bioaccumulated between Day 0 and Day 8 and reached a plateau after the 9‐day intake period. This observation aligns with previous pharmacokinetic studies, which demonstrated that, while a linear relationship between vitamin B6 intake and blood levels of PLP is observed for doses up to 8 mg/day [45, 46], higher intakes did not show a dose‐response progression, and PLP levels reached an upper level steady‐state threshold [47].

The use of the NMES model with forced lengthening contractions has been confirmed to trigger myofiber necrosis and elicit a robust response from MuSCs, leading to the appearance of regenerating fibers. However, the degree of myofiber injury damage varied significantly among participants. This is likely influenced by individual tolerability to the electrical stimulation and inter‐individual variation in the properties of the subcutaneous tissues. Furthermore, human studies rely on small muscle biopsies, which may not capture the full extent of the injury. This important variability poses a challenge in normalizing MuSCs and their progeny. Considering the strong correlation between muscle damage and regeneration, it was crucial to account for the degree of damage to better estimate the potential treatment effects.

NAM, as other forms of vitamin B3, can be converted to NAD^+^, an essential coenzyme for redox reactions central to energy metabolism, and a cofactor or substrate of hundreds of enzymes playing a role in multiple cellular processes [48]. In our previous preclinical research [31], we have discovered that the effect of NAM on MuSCs was independent of NAD^+^ as itdid not require its conversion to NAD^+^, and signaled through a partial CK1‐mediated β‐catenin activation. Several NAD^+^ precursors can directly or indirectly generate NAM in vivo after oral intake [49]. In recent years, a few clinical trials have investigated the effects of different NAD^+^ precursors on skeletal muscle homeostasis and regeneration. One notable study by Jensen et al. explored the impact of nicotinamide riboside (NR) supplementation, in combination with pterostilbene, on muscle regeneration in elderly individuals [50]. Their findings, primarily evaluating MuSC content measured by flow cytometry, did not demonstrate any significant effect on muscle regeneration. It is important to note that the study by Jensen et al. lacked a direct marker for assessing the extent of muscle damage, which limited their ability to evaluate the isolated effect on damaged fibers. Furthermore, the lower blood creatine kinase levels observed 2 days after the contraction protocol in their study (approximately an average of 1000 U/L) compared to our study (approximately 6000 U/L) suggest that the extent of myofiber damage was less substantial. Another study investigated the long‐term effects of NR supplementation on mitochondrial biogenesis in muscle and fat tissue [51] reported a beneficial effect on MuSC differentiation showing a change in the ratio of Pax7 expression over the late myogenic differentiation marker myogenin. However, this study did not involve muscle injury and MuSC readouts were exploratory and mostly deduced from gene expression analyses on whole muscle or cultured myoblasts. Overall, direct comparisons with the studies by Jensen et al. and Lapatto et al. are precluded due to differences in supplementation approaches and variations in evaluation methods. Moreover, we have shown in a mouse model that the levels of NAM resulting from NR conversion were 50 times lower compared to the levels achieved through direct NAM supplementation, failing to reach the threshold at which NAM exhibits its active effects on MuSCs [31]. Additionally, the MuSC and muscle repair efficacy in the present study not only relies on NAM but on the combination of NAM with PN [31]. We have indeed previously demonstrated in our preclinical studies [31] that PN enhances the differentiation of Pax7^+^ human myogenic progenitors to MyoD^+^, independent of muscle stem cell proliferation. We showed that this mechanism requires PI3K/AKT as it both activates this signaling pathway and the stem cell response can be blocked by PI3K inhibition. Moreover, conversion of PN to its bioactive form PLP also catalyzes several rate‐limiting reactions for energy metabolism [52], which may also contribute to myogenic fate directly or synergize with AKT signaling as also reported in other tissues and species [53, 54, 55]. Further analyses will be important to understand if PN activates PI3K/AKT signaling via a specific molecular target or enhances AKT signaling and myogenic differentiation secondary to metabolic adaptations via PLP‐sensitive enzymes.

Currently, there are no treatments specifically targeting MuSCs for promoting muscle repair and recovery that are implemented in clinical practice. Most of the available experimental solutions are at the preclinical stage, with only a limited number of clinical studies conducted. Notably, testosterone has been shown to stimulate MuSC proliferation in humans [56, 57], but it is not without side effects and safety considerations are crucial when developing treatment approaches for promoting muscle regeneration across different age groups and severity of cases. Nutritional solutions may offer a safer solution. The role of dietary protein in supporting muscle adaptation and growth following exercise in adults has been demonstrated [58, 59]. This beneficial effect has been primarily attributed to the well‐established impact on myofibrillar protein synthesis [60, 61] with few studies also demonstrating a potential benefit on MuSCs [62, 63]. Therefore, our findings on the positive effect of NAM/PN supplementation on MuSCs and myofiber regeneration have direct opportunities for clinical translation for the management of sport‐related muscle damage, where boosting regeneration can accelerate the repair process and could be combined with dietary protein strategies for enhanced muscle recovery. In this context it is worth noting, given the capacity for myofibers to repair minor damage through myonuclear‐mediated processes independent of MuSCs [64], that NAM/PN supplementation will be most effective where MuSCs are involved. Furthermore, while the effects of NAM/PN on MuSCs were analyzed in this study independent of strength training, which was an exclusion criteria given its protective role on muscle repair [65], it is possible that regular physical activity may cross‐talk with the effects of NAM/PN on muscle stem cells. Since physical activity was not measured in this acute study, longer studies with physical activity monitoring will be required to answer this question.

Our findings could also have implications for a wide range of muscle disorders in which compromised MuSC function and muscle regeneration are pathological hallmarks. Notably, sarcopenia, the age‐related loss of muscle mass, strength, and function, is in part characterized by a decline in MuSC function and number, resulting in compromised regenerative capacity of skeletal muscle [24, 66, 67, 68, 69]. A clinical study has shown that older individuals struggle to recover from disuse‐induced atrophy and fail to expand the MuSC pool during rehabilitation [70]. Our previous preclinical investigations including rodent models and primary myoblasts from aged humans have demonstrated that NAM/PN overcomes MuSC dysfunction and regenerative failure associated with aging [31]. Taken together, these findings strongly support the potential of NAM/PN as a novel nutritional intervention for alleviating age‐related muscle decline. Another application is cancer cachexia which results from systemic inflammation and an imbalance between protein synthesis and degradation [71], coupled to myofiber damage and impaired regeneration in the muscle wasting process [20, 21, 72]. Interestingly, using mouse models of cancer cachexia, two studies have shown that supplementation with niacin could mitigate muscle wasting [73, 74] but the potential role of MuSCs was not investigated. Muscular dystrophies and myopathies encompass a wide and heterogeneous group of genetic conditions characterized by muscle weakness [75]. Altered MuSC function and muscle regenerative capacity are common hallmarks of several muscular dystrophies and myopathies [76]. Notably, targeting MuSCs in the context of Duchenne Muscular Dystrophy (DMD) holds great potential in mitigating the dystrophic phenotype [77, 78, 79]. Several pharmacological drugs that affect MuSC function have demonstrated efficacy in preclinical models of DMD [80, 81, 82]. Additionally, while MuSCs play a central role in muscle regeneration, muscle maintenance and repair require a highly orchestrated coordination between multiple cell types and their microenvironment [83]. For example, MuSCs have been shown to secrete exosomes that regulate collagen biosynthesis in fibrogenic cells to prevent excessive extracellular matrix deposition [84]. Fibrosis being a hallmark of many muscle diseases, these results highlight a potential benefit of promoting MuSC function beyond the formation of healthy myofibers [14].

Although our study did not directly investigate these specific disorders, the observed positive effects of NAM/PN supplementation in healthy volunteers may have potential benefits for individuals affected by these conditions. Further research is needed to explore the specific mechanisms and implications of NAM/PN supplementation in muscle disorders. It should be noted that this study was conducted exclusively in men for standardization purposes. Although we do not anticipate that the findings would differ in women as we have seen that NAM/PN activates the number of Pax7‐positive and MyoD‐positive primary human myogenic progenitors from female donors ex vivo (Ancel et al., JCI 2024), it will be important to include women in future studies testing the efficacy of NAM/PN.

## 4 Materials and Methods

### 4.1 Study Design and Participants

This study was approved by the Regional Scientific Ethical Committees of Copenhagen (protocol number H‐20081080). All participants provided written consent to participate, after the nature and possible consequences of the studies were explained, and all procedures conformed to the Declaration of Helsinki. The study was executed as a single‐center, randomized, double‐blind, placebo‐controlled trial and conducted in the Institute of Sports Medicine Copenhagen between April 2021 and May 2022. The study was pre‐registered on ClinicalTrials.gov with the identifier NCT04874662 and the number of muscle stem cells as primary endpoint. Sample size calculation was calculated on the primary endpoint based on a variability of 20% [29] and an effect size of 30% inferred from preclinical models [31]. To reach a statistical power of 80%, if all the assumptions made were met, 20 participants were needed per group (40 participants in total).

Before inclusion all participants were screened by a physician and deemed overall healthy based on blood samples, urine analysis, blood pressure measurement, EKG, neurological exam, medical history and an interview. All participants were men, aged between 18–49 with a BMI between 18.5 and 24.9 kg/m^2^ and had normal dietary habits. Exclusion criteria included current smoking, participation in structured strength exercise less than 3 months prior to participation in the study, use of corticosteroids, anabolic steroids, growth hormones, anti‐coagulant or anti‐aggreging agents or having participated in a clinical study in the last 3 months. Consumption of supplements containing vitamin B3 and/or vitamin B6 was also prohibited at least 2 weeks before the inclusion and during the intervention, other than those provided by the investigator. Eligible participants were randomized in a 1:1 ratio to supplementation (NAM/PN) or control group (placebo), on Day 0 by an investigator not involved in the inclusion process. The randomization was stratified by age (18≤age<35 and 35≤age≤50). Inclusion of participants from both groups was similarly distributed throughout the study period.

### 4.2 NAM/PN and Placebo Administration

Participants received, in a double‐blinded manner, oral supplementation with NAM/PN (714 mg NAM + 19 mg PN, DSM Nutritional Products GmbH, Germany) or placebo (microcrystalline cellulose, Hänseler AG, Herisau, Switzerland) as two capsules per day to be taken in the morning in fasting (overnight sleep) condition for 9 days (from Day 0 to Day 8). The first dose was taken prior to performing the muscle injury protocol. Doses of NAM and PN were selected based on cellular efficacy in human myogenic progenitors [31] and a combination of mouse in vivo oral studies, human dose modeling based on published pharmacokinetic studies, allometric scaling and benchmarking to toxicology and approved regulatory limits.

### 4.3 Research Objectives and Prospective Selection of Endpoints

The primary objective of this study was to investigate if a combination of nicotinamide (NAM) and pyridoxine (PN) intake would improve human skeletal muscle regeneration, through enhanced activation of MuSCs. As stated in the trial registration (NCT04874662), the primary endpoint was prospectively selected as the number of MuSCs (Pax7 and myogenin) on sections of muscle biopsies from the stimulated leg, comparing the study intervention (NAM/PN) to the control intervention (placebo).

### 4.4 Muscle Injury Protocol

One leg, dominant or non‐dominant, was randomly selected for neuromuscular electrical muscle stimulation (NMES) coupled with eccentric contractions to induce muscle damage. Notably, no statistically significant evidence (p<0.05) was found that leg dominance influences the overall response to the exercise protocol, nor that the observed group differences are explained by leg dominance (Table S1). The electrical stimulation was applied to the vastus lateralis with a CefarCompex mi‐Theta 600 Muscle stimulator (DJO Nordic, Malmö, Sweden). Adhesive electrodes were placed on shaved and cleaned skin. A 10×5 cm adhesive electrode was placed proximal (inguinal area) on the vastus lateralis. And two 5×5 cm adhesive electrodes were placed distally on the vastus lateralis, spaced out so the entire width of the vastus lateralis was covered. After placements of the adhesive electrodes the participants did a 5 min warm up of slow to moderate cycling on a bike ergometer, followed by 5–10 min familiarization with the electric stimulation, where the electric current was gradually increased. The stimulation was delivered every 15 s with a frequency of 35 Hz, 300 µs pulse‐duration, 0–120 mA, and a 0,75 s ramp‐up/down period.

The stimulations were performed in a Kin‐Com dynamometer (Kin‐COM, model 500–11, Kinetic Communicator, Chattecx, Chattanooga, TN), in which each stimulation was accompanied by mechanical flexion of the knee. A total of 5×20 slow (3.5 s stimulations and flexion with an angular velocity of 30° pr. second) and 5×20 fast (1.5 s stimulation and flexion with an angular velocity of 180° pr. second) stimulations. The stimulation current was continuously increased till the participants tolerations threshold. This method was slightly adapted from previous used protocols [25, 29].

### 4.5 Blood Samples

Blood samples were collected at Day 0, Day 2, Day 4 and Day 8 from an antecubital vein into EDTA tubes and placed on ice until storage at −80°C. Blood analysis of creatine kinase was carried out at the Clinical Biochemistry department at Bispebjerg and Frederiksberg Hospital.

### 4.6 Pharmacokinetic Assessments

NAM, PN and related metabolites pharmacokinetic profiles were assessed on blood samples at Day 0 and Day 8. For each day, blood will be collected at four different timepoints (pre‐supplementation, and 30 min, 1 h, and 3 h post‐supplementation). Participants were in fasting conditions until the 3 h post‐supplementation timepoint for blood collection. Serum was rapidly processed and stored at −80°C. Concentrations of nicotinamide (NAM), pyridoxal 5’‐phosphate (PLP, bioactive from of PN) and pyridoxine (PN) were measured using LC‐MS/MS by BEVITAL (Bergen, Norway; www.bevital.no). Measurements were performed by mixing samples with labelled internal standards, separation on a C8 liquid chromatography column by a gradient‐type mobile phase and detecting analytes using electrospray ionization tandem mass spectrometry as described in [85].

### 4.7 Muscle Biopsies

Skeletal muscle biopsies were obtained from the vastus lateralis muscles using the percutaneous needle biopsy technique of Bergström [86]. 1% lidocaine (amgros I/S, Copenhagen, Denmark) was applied subcutaneously as local anesthetic, and tissue was extracted with a 5 mm diameter biopsy needle and manual suction. The biopsies were spaced by a minimum of 2 cm. Each biopsy was divided with one main portion being embedded in Tissue‐Tek (Sakura Finetek Europe, Zoeterwoude, the Netherlands) and frozen in isopentane, precooled by liquid nitrogen for cryosectioning and immunohistofluorescence analyses and the remaining smaller portion being snap frozen in liquid nitrogen. All muscle tissue was stored at −80°C.

### 4.8 Muscle Soreness

Muscle soreness was evaluated using a visual analogue scale (VAS), which ranges from 0 (normal, no pain) to 10 (extremely painful). Participants were instructed to slowly sit down unassisted (5 s) onto a chair, at home, first thing in the morning, and note on a scale of 1–10 how painful the front thigh muscles (of the stimulated leg) were during this movement. Soreness was daily assessed starting the morning before muscle injury until Day 8.

### 4.9 Muscle Strength Test

Maximal voluntary isometric strength (MVIC) was tested by measuring peak isometric torque at a 70° knee flexion in an isokinetic dynamometer (Kinetic Communicator model 500–11). The best of three attempts was reported. The tests were performed immediately before and after the injury protocol, as well as on Day 2, Day 4 and Day 8. This test measures the strength of the whole quadriceps muscle which consists of four individual muscles. It is used as an early marker of muscle damage to assess the effectiveness of the injury protocol, but its sensitivity is limited because only one out of the four muscles involved in the strength test is subjected to electrical stimulation and thus damaged (vastus lateralis).

### 4.10 Immunohistofluorescence

Frozen tissues were sectioned at 10 µm with a cryostat (Leica Biosystems) at −20 °, and sections were placed on glass slides (Superfrost Plus), which were stored at −80°C before being immunolabeled.

All biopsy timepoints were immunolabeled for Pax7 (DSHB, Pax7, supernatant, 1:100), myogenin (DSHB, F5D, supernatant, 1:100), eMyHC (DSHB, F1.652, supernatant, 1:100) and dystrophin (Sigma, D8168, 1:100), each one in combination with laminin (Sigma, L9393, 1:200). Primary antibodies were applied overnight to fixed (myogenin; Histofix, 10 min), or unfixed (dystrophin, eMyHC), sections according to the respective antibody datasheets. Sections were then incubated in a cocktail of two secondary antibodies (Thermo Fischer Scientific A‐11029, A‐11036) for 45 min before mounting in ProLong Gold Antifade Reagent, containing DAPI (Invitrogen, P36931). Dystrophin and eMyHC sections were fixed (Histofix, 10 min) before mounting. For Pax7, sections were fixed in 4% PFA (5 mins) before overnight incubation with antibodies against Pax7, laminin and myosin heavy chain I (DSHB, BA‐D5, 1:00), visualized by three secondary antibodies (Thermo Fischer Scientific A‐21121, A‐21144, A‐21076). The DSHB Hybridoma products F1.652, F5D, Pax7, and BA‐D5 were deposited to the DSHB by H. M. Blau, W. E. Wright, A. Kawakami, S. Schiaffino, respectively. These sections were imaged with the AxioScan 7 or Z1 slide scanner and images were analyzed by manually counting positive (or, for dystrophin, negative) cells in QuPath.

For a more detailed analysis of the Day 8 samples, Pax7/MyoD/dystrophin co‐immunolabelings and myogenin/dystrophin co‐immunolabelings, slides were performed with various combinations of antibodies against laminin (Sigma, L9393, 1:1000), dystrophin (Sigma, D8168, 1:500) and Pax7 (DSHB, purified, 2.5 µg/ml, 1:250); MyoD (Abcam, ab133627, 1:500); myogenin (Abcam, ab124800, 1:500); and Hoechst 33342 (Sigma, B2261). All antibodies are diluted in blocking buffer containing 4% BSA (Jackson ImmunoResearch, 001‐000‐162) in PBS. Slides were fixed with 4% PFA (EMS, #157‐4‐100), for 15 min and permeabilized in cold methanol (VWR, #1.06035.2500) for 6 min. Antigen retrieval was performed with two successive incubations of hot 0.01 M pH 6 citric acid during 4 min, and sections were further blocked in 4% BSA for 2 h. To avoid technical issues like cross reaction, interaction, nuclear markers were immunolabeled and detected first. Slides were sequentially incubated overnight with primary antibodies anti‐Pax7, anti‐MyoD, or anti‐myogenin at 4°C. Before new incubation, slides were washed three times in PBS/0.1% Triton X‐100. Pax7 signal was further amplified using a goat‐anti mouse IgG1‐biotin (Jackson ImmunoResearch, 115‐065‐205, 1:1000) followed by conjugation with Streptavidin Alexa555 (Life Technologies, #S‐21381, 1:2000), and other antibodies were detected with their specific secondary antibodies (Thermo Fisher Scientific, A32733), while nuclei were detected with Hoechst 33342 (1:10 000). Then, the slides were immunolabeled for fiber structures. Slides were incubated with primary antibodies anti‐ laminin, anti‐dystrophin for 3 h at room temperature. Finally, primary antibodies were detected using anti‐rabbit IgG (Life Technologies, A‐21039, 1:2000) and anti‐mouse IgG2b (Life Technologies, A‐21141, 1:2000).

For eMyHC/dystrophin co‐immunolabeling and IgG immunolabeling, slides were treated with various combinations of antibodies against laminin (Sigma, L9393, 1:1000), dystrophin (Sigma, D8168, 1:500), human IgG (Beckman Coulter, A79391) and eMyHC (DSHB, F1.652, 1:500); and Hoechst 33342 (Sigma, B2261). All antibodies are diluted in blocking buffer containing 4% BSA (Jackson ImmunoResearch, 001‐000‐162) in PBS. After 15 min of rehydration in PBS, slides were blocked for 45 min at room temperature in 4% BSA. Slides were immunolabeled for 3 h at room temperature using anti‐laminin and anti‐eMyHC antibodies. Sections were then incubated for 1 h with secondary antibodies (Thermo Fisher Scientific, A‐21127, A‐21039, A‐ 21127, 1:2000) at room temperature and counterstained with Hoechst (1:10 000). Then, dystrophin immunolabeling was performed as described previously. All slides were then mounted using Dako fluorescent mounting medium (Agilent, #S302380‐2). The size of myofibers was calculated across the entire section on all fibers using an automated image processing algorithm developed internally using the QuPath software and the Fiji open‐CSAM tool [87].

### 4.11 Imaging and Quantification

All biopsy sections were entirely scanned with an Olympus VS200 slide scanner at 20× magnification and stitched to 1 image. Quantifications of Pax7^+^, MyoD^+^, myogenin^+^ cells were determined by manually counting on entire muscle sections with researchers blinded to the experimental groups. Quiescent Pax7^+^ cells were distinguished as Pax7^+^ cells belonging to dystrophin‐positive fibers and residing under the basal lamina whereas activated Pax7^+^ cells were distinguished as being located inside or in close proximity to dystrophin‐negative fibers.

Fibers were detected across the entire sections by an automated image processing algorithm developed internally using the QuPath software and the Fiji open‐CSAM tool. Fibers are detected and classified based on both dystrophin and laminin immunolabeling. A quality control was performed to remove artifacts. Missing fibers were manually drawn and assigned to the correct class. The number of uninjured fibers was determined from the sections as being dystrophin‐ and laminin‐positive, whereas damaged fibers were detected as being laminin‐positive and dystrophin‐negative. To quantify the extent of myofiber regeneration, sections were immunolabeled for embryonic myosin heavy chain (eMyHC). After cross sectional analysis, the total number of regenerating fiber (eMyHC^+^) and the area covered by eMyHC into the damaged fibers were quantified by applying a threshold analysis to the whole image via Qupath software (pixel classification option).

### 4.12 Statistics

All data were analyzed and plotted using R version 4.2.2 and GraphPad Prism 9.0 software. Normality of data was determined using Shapiro Wilk normality test. Comparisons were performed using Welch's t‐test for normally distributed data or Wilcoxon rank sum test (equivalent to the Mann‐Whitney test) for non‐normally distributed data, corresponding paired tests were performed whenever appropriate. Normally distributed data are represented as mean ± SEM and non‐normally distributed data as median with interquartile range. Multiple comparisons were performed using a Kruskal‐Wallis test with Dunn's post hoc test or a Friedman test followed by pairwise Wilcoxon signed rank tests to identify different groups and Bonferroni corrections for multiple testing. Spearman's correlation analyses were conducted for associations between continuous variables. The per‐protocol analysis comprised all 39 participants who completed the study.

Because activated MuSCs and muscle regeneration cannot be quantified in the absence of muscle damage, a subgroup statistical analysis was conducted with exclusion of participants with very low muscle damage. Very low muscle damage was defined as the first quartile of the dystrophin‐negative fiber distribution, corresponding to 0.74%–1.38% of dystrophin‐negative fibers (for each readout the sample size is indicated in the respective figure legend). Raw data for the number of fibers, dystrophin‐negative fibers, quiescent Pax7^+^ cells, activated Pax7^+^ cells, MyoD^+^ cells and eMyHC^+^ fibers are presented in Table 3. All statistical analyses were 2 tailed, and *p* values of 0.05 or less were considered statistically significant.

**TABLE 3:** Raw data for the number of fibers, dystrophin‐negative fibers, quiescent Pax7^+^ cells, activated Pax7^+^ cells, MyoD^+^ cells and eMyHC^+^ fibers.

			Placebo group					NAM/PN group				
			Average	SD	Median	Min	Max	Average	SD	Median	Min	Max
Pax7/MyoD analyses	Per‐protocol population	Total fibers	1225	458	1090	509	2099	987	477	913	324	2063
		Dystrophin‐negative fibers	243	255	150	5	830	151	204	43	5	639
	Subjects with apparent muscle damage (>first quartile based on % dystrophin‐negative fibers)	Total fibers	1165	354	1086	753	2014	1004	517	973	324	2063
		Dystrophin‐negative fibers	321	249	196	40	830	203	216	82	21	639
Myogenin analyses	Per‐protocol population	Total fibers	1134	469	977	500	2034	926	409	885	280	1819
		Dystrophin‐negative fibers	218	224	119	0	687	130	174	43	0	514
	Subjects with apparent muscle damage (>first quartile based on % dystrophin‐negative fibers)	Total fibers	1039	327	951	630	1874	931	448	905	280	1819
		Dystrophin‐negative fibers	288	217	246	29	687	175	183	71	10	514
eMyHC analyses	Per‐protocol population	Total fibers	1188	428	988	660	1925	973	452	975	290	1884
		Dystrophin‐negative fibers	233	247	124	4	755	137	185	46	1	560
	Subjects with apparent muscle damage (>first quartile based on % dystrophin‐negative fibers)	Total fibers	1107	380	971	660	1925	964	499	975	290	1884
		Dystrophin‐negative fibers	290	245	193	23	755	197	198	73	11	560

Per‐protocol population												
			Placebo group					NAM/PN group				
			Average	SD	Median	Min	Max	Average	SD	Median	Min	Max
	Pax7/MyoD analyses	quiescent Pax7+ cells	169	70	154	82	293	157	70	165	61	277
		activated Pax7+ cells	112	137	57	3	513	92	133	18	2	457
		MyoD+ cells	343	387	172	8	1203	260	315	110	14	949
	Myogenin analyses	Myogenin+ cells	359	409	159	7	1305	241	314	73	4	1043
	eMyHC analyses	eMyHC+ fibers	130	174	35	0	610	107	157	24	0	425

Subjects with apparent muscle damage (>first quartile based on % dystrophin‐negative fibers)												
			Placebo group					NAM/PN group				
			Average	SD	Median	Min	Max	Average	SD	Median	Min	Max
	Pax7/MyoD analyses	quiescent Pax7+ cells	165	71	159	82	293	165	77	173	61	277
		activated Pax7+ cells	148	141	96	14	513	124	143	43	10	457
		MyoD+ cells	449	394	191	26	1203	346	327	227	53	949
	Myogenin analyses	Myogenin+ cells	474	413	274	67	1305	322	331	163	43	1043
	eMyHC analyses	eMyHC+ fibers	163	181	82	2	610	153	169	45	5	425

## Author Contributions

Designed and experimental strategy: GH, JM, LGK, MK, JNF, PS, and ALM. Interpretation of results, and writing the manuscript: GH, JM, JNF, PS, and ALM. Performed experiments: GH, JM, AD, KK, SK, BWH, WH. Contributed to experimental strategy and data interpretation: MK, EPM, OEJ, LGK. Analyzed data: EM, GH, JM, JNF, PS, ALM. Conceived and lead the project: JNF, PS. Co‐first authors share primary responsibility in conducting experiments, analyses, and interpretation of results for this study. The order of co‐first authors reflects the contribution to writing and editing of the manuscript: GH, JM. All authors read and approved the final manuscript.

## Funding

This study was funded by Nestlé, with support from: Lundbeck Foundation grant R344‐2020–254 (to ALM) BRIDGE — Translational Excellence Programme (bridge.ku.dk) at the Faculty of Health and Medical Sciences, University of Copenhagen, funded by the Novo Nordisk Foundation (NNF20SA0064340) (to GH).

## Trial Registration

NCT04874662.

## Conflicts of Interest

J.M., E.M., S.K., J.N.F., and P.S. are employees of Société des Produits Nestlé SA; E.P.M., and O.E.J. are employees of Nestlé Health Science SA. L.G.K. was an employee of Nestlé Health Science SA and is on the scientific advisory boards of Vital Proteins and NUUN, has participated on advisory boards of Liquid I.V and has received personal fees from RNWY and Nestle Health Science; is a board member of Siftlink. G.H., A.D., K.K. B.W.H., W.H., M.K., and A.M. declare that they have no competing interests.

## Supporting information




**Supporting File**: advs74747‐sup‐0001‐SuppMat.docx.

## Data Availability

All data are available in the main text or the supplementary materials. Raw data are provided in Supplemental File S1.
