# Supplementary material for: Nicotinamide and Pyridoxine Supplementation Enhances Muscle Stem Cell Activity and Muscle Regeneration in Humans: A Randomized Placebo‐Controlled Clinical Trial of High Force Eccentric Contraction Recovery in Healthy Young Men
Source: Adv Sci (Weinh). 2026 Mar 24;13(28):e18471. doi: 10.1002/advs.202518471 (PMC13185830; doi:10.1002/advs.202518471)
Supplement: Supplementary file 1 — Supporting File: advs74747‐sup‐0001‐SuppMat.docx. [file ADVS-13-e18471-s001.docx]

**
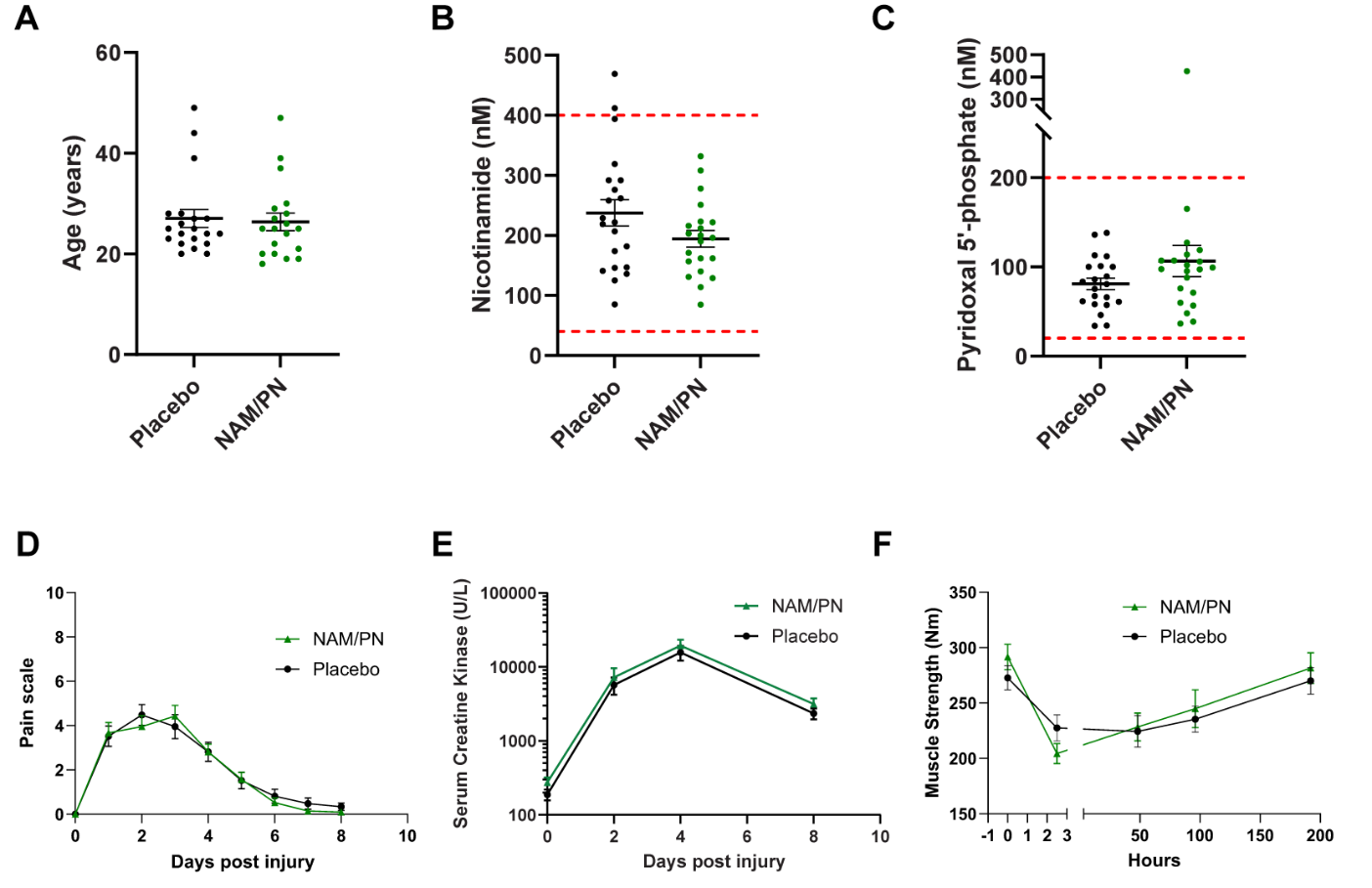
**

**Figure S1: Study design, basal levels of NAM/PN and evaluation of the Neuromuscular Electrical stimulation (NMES) protocol.** (A) Age of participants in years at the time of enrollment. (B,C) Blood levels of (B) nicotinamide and (C) pyridoxal 5’-phosphate (bioactive form of pyridoxine), at baseline (Day 0, pre-supplementation). Red dotted lines indicating low and high references values. (D) Delayed onset muscle soreness (or muscle soreness) was evaluated using a visual analogue scale, which ranges from 0 (normal, no pain) to 10 (extremely painful), at baseline (D0, pre-NMES) and after having induced muscle damage (daily at home by the study participants from Day 1 to Day 8). (E) Serum creatine kinase measurement in blood samples collected for the hematology and serum chemistry assessment at Day 0, Day 2, Day 4 and Day 8. (F) Muscle strength was recorded at baseline (Day 0, pre-NMES) and after having induced muscle damage at Day 0 + 2.5hrs, Day 2, Day 4 and Day 8 in the stimulated leg. Maximal isometric muscle strength was measured in a seated position in a dynamometer at a 70° knee angle (straight leg is zero degrees), as the greatest knee extensor torque measured over 3 attempts. Scale bars = 100µm.

**
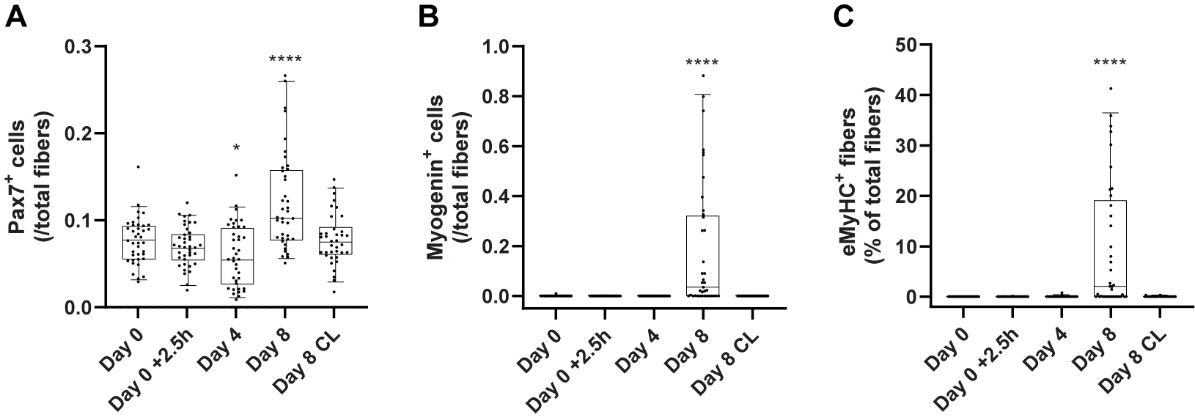
**

**Figure S2: NMES protocol induces a regenerative response.** (A-C) Content of Muscle Stem Cell (MuSC) and their progenies were assessed by immunohistofluorescence on cross sections of muscle biopsies collected at Day 0 (pre-NMES, contralateral leg) and at Day 0 + 2.5hrs (stimulated leg), Day 4 (stimulated leg) and Day 8 (stimulated leg and contralateral leg (CL)) after having induced a muscle injury. (A) Activation and proliferation of MuSCs was evaluated by the quantification of Pax7^+^ cells. (B) Differentiation of MuSCs was measured by the number of myogenin^+^ cells and (C) fiber regeneration was assessed by the proportion of fibers displaying immunoreactivity for embryonic myosin (eMyHC). Data are expressed relative to the total number of fibers with individual dots representing individual participants (n=39). *, **** indicated significant difference (p<0.05, p<0.0001, respectively) compared to baseline (Day 0).


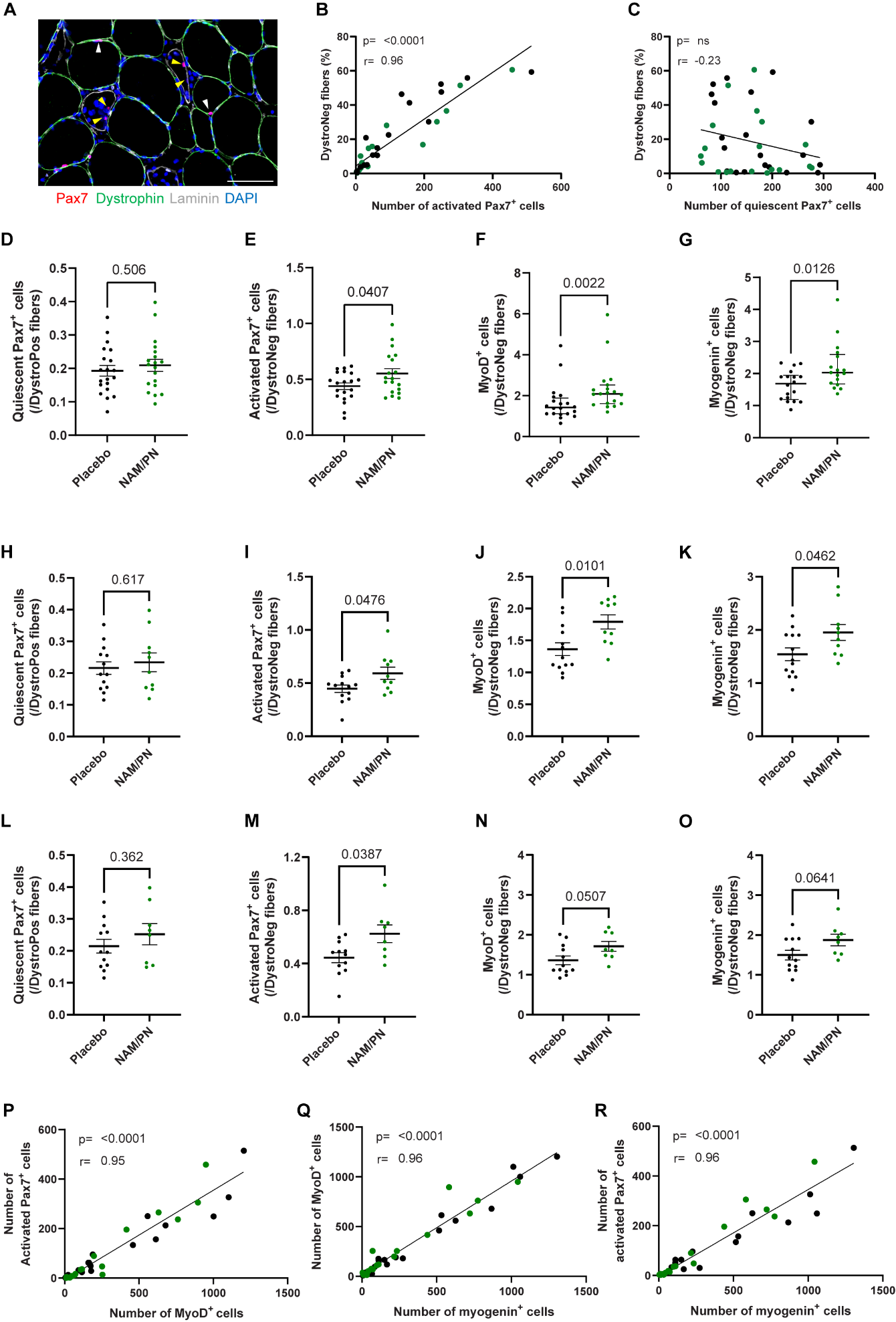


**Figure S3:** **NAM/PN-induced MuSC activation (activated Pax7^+^, MyoD^+^ and myogenin^+^ cells) persist across independent stratifications of muscle damage severity.** (A) Quiescent vs. activated Pax7^+^ cells were distinguished as mononucleated cells belonging to dystrophin-positive fibers and residing under the basal lamina (indicated by white arrowheads) whereas activated Pax7^+^ cells were distinguished as being located inside or in close proximity of dystrophin-negative fibers (indicated by yellow arrowheads). Scale bar = 100µm. (B, C) Correlation analyses (placebo group, n=20; NAM/PN group, n=19) between the percentage of dystrophin-negative fibers and the number of (B)activated Pax7^+^ cells, (C) quiescent Pax7^+^ cells. (D-O) The number of MuSCs and their progenies were quantified by immunohistofluorescence on cross section of biopsies collected at Day 8 (stimulated leg). Analyses were performed on three different sets of subjects: (D-G) all participants from per-protocol population (placebo group, n=20; NAM/PN group, n=19); (H-K) participants with 5% or more of dystrophin-negative fibers (placebo group, n=13; NAM/PN group, n=10); (L-O) participants at or above the median value for the percentage of dystrophin-negative fibers (placebo group, n=12; NAM/PN group, n=8). Comparison between NAM/PN vs. placebo group of the number of (D, H, L) quiescent Pax7^+^ cells, (E, I, M) activated Pax7^+^ cells, (F, J, N) MyoD^+^ cells, and (G, K, O) myogenin^+^ cells. Data are represented as (D,E, H-O) mean ± SEM or (F,G) median with interquartile range, and are expressed relative to the number of (D, H, L) dystrophin-positive (DystroPos) and to the number of (E-G; I-K, M-O) dystrophin-negative (DystroNeg) fibers. (P-R) Correlation analyses between the number of (P) activated Pax7^+^ cells and the number of MyoD^+^ cells, between the number of (Q) MyoD^+^ cells and the number of myogenin^+^ cells and between the number of (R) activated Pax7^+^ cells and the number of myogenin^+^ cells. Placebo group, n=20; NAM/PN group, n=19. Individual dots represent individual participants from the Placebo group (black dots) and the NAM/PN group (green dots).


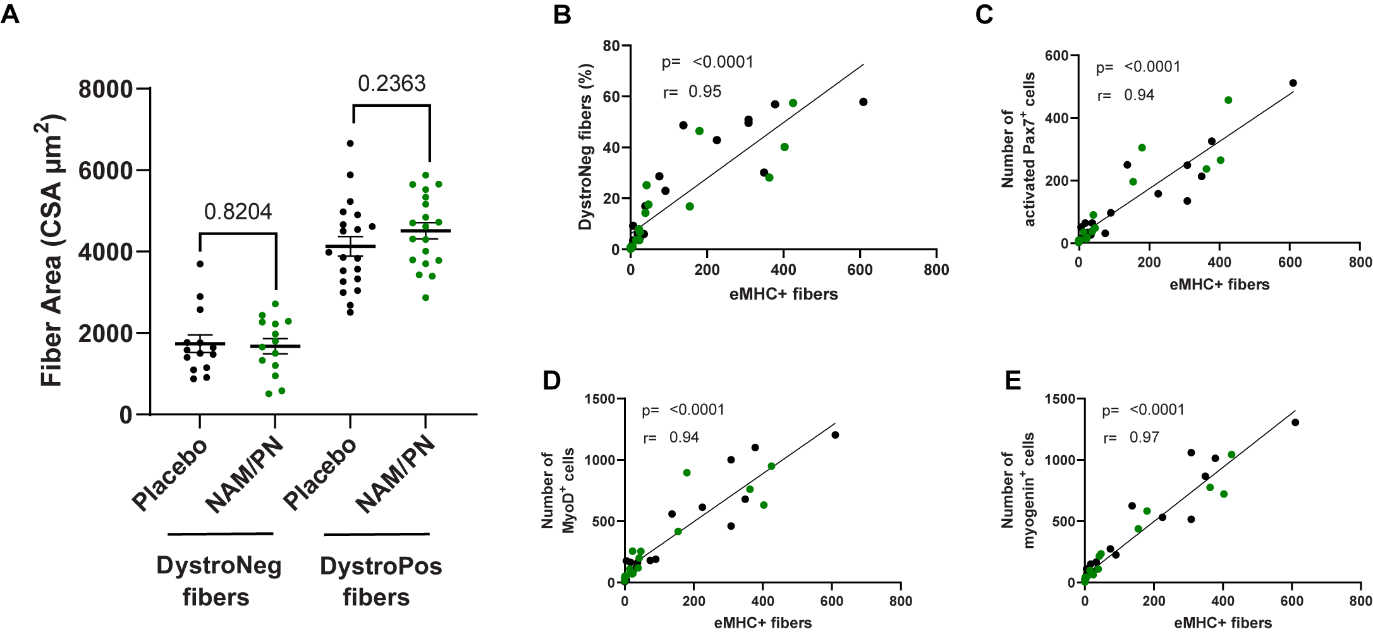


**Figure S4: Evaluation of fiber regeneration by immunohistofluorescence on cross sections of biopsies collected at Day 8 (stimulated leg). (A)** Quantification of myofiber cross-sectional area (CSA). (B) Correlation analysis between the percentage of dystrophin-negative fibers and the number of eMyHC^+^ fibers. (C-E) Correlation analyses between the number of eMyHC^+^ fibers and the number of (C) activated Pax7^+^ cells, (D) MyoD^+^ cells and (E) myogenin^+^ cells. Individual dots represent individual participants from the Placebo group (black dots, n=20) and the NAM/PN group (green dots, n=19).

**Table S1.** Output of linear models to estimate the effect of leg dominance.
